# A Novel Mutation in Sacsin, p.Val1335IIe, May Cause Late-Onset Sacsinopathy Due to Haploinsufficiency

**DOI:** 10.3390/cimb45120619

**Published:** 2023-12-09

**Authors:** Danyeong Kim, Nayoung Ryoo, Young Ho Park, Eva Bagyinszky, Seong Soo Alexander An, SangYun Kim

**Affiliations:** 1 Department of Bionano Technology, Gachon University, Seongnam 13120, Republic of Korea; dan627328@gmail.com; 2 Department of Neurology, Eunpyeong St. Mary’s Hospital, College of Medicine, The Catholic University of Korea, Seoul 03083, Republic of Korea; nyryoo@gmail.com; 3 Department of Neurology, Seoul National University College of Medicine, Seoul National University Bundang Hospital, Seongnam 13620, Republic of Korea; kumimesy@snubh.org; 4 Graduate School of Industrial and Environmental Engineering, Gachon University, Seongnam 13120, Republic of Korea; navigator120@gmail.com

**Keywords:** sacsinopathy, *SACS*, ARSACS

## Abstract

Autosomal recessive spastic ataxia in Charlevoix-Saguenay (ARSACS) is a neurodegenerative disorder caused by mutations in the sacsin molecular chaperone protein (*SACS*) gene. Since the first report from Quebec in 1978, many pathogenic ARSACS variants with significantly reduced chaperone activities have been reported worldwide in adolescents, with presumably altered protein folding. In this study, a novel *SACS* mutation (p.Val1335IIe, Heterozygous) was identified in a Korean patient in their 50s with late-onset ARSACS characterized by cerebellar ataxia and spasticity without peripheral neuropathy. The mutation was confirmed via whole exome sequencing and Sanger sequencing and was predicted to likely cause disease using prediction software. RT-PCR and ELISA showed decreased *SACS* mRNA expression and sacsin protein concentrations in the proband, supporting its implications in diseases with pathogenicity and reduced chaperone function from haploinsufficiency. Our results revealed the pathogenicity of the *SACS* Val1335IIe mutation in the proband patient’s disease manifestation, even though the symptoms had a limited correlation with the typical ARSACS clinical triad, which could be due to the reduced chaperon function from haploinsufficiency. Furthermore, our study suggests that variants of *SACS* heterozygosity may have diverse symptoms, with a wide range of disease onsets for late-onset sacsinopathy.

## 1. Introduction

Since the first report of autosomal recessive spastic ataxia of Charlevoix-Saguenay (ARSACS) in Quebec [1,2], several cases have been reported worldwide, including Italy, the Netherlands, Brazil, Belgium, and Japan [3]. In Korea, three cases of ARSACS have been reported in young patients [4,5,6]. Clinically, ARSACS usually starts at an early age, mostly before 13 years of age, with symptomatic presentations of three clinical triads: slowly progressive cerebellar ataxia, limb spasticity, and peripheral neuropathy. However, with an increasing number of patients worldwide, atypical clinical features have been reported, including hypermyelination of the retina, nystagmus, amyotrophy (muscle wasting), dysarthria, dysphagia (swallowing difficulty), pes cavus (high-arched feet), scoliosis (a spine that curves to the side), urinary tract problems, hearing loss, and recurrent seizures [7,8,9,10]. What became clear after gene identification was that classical features were not always present, that patients carrying the same genotype had variable phenotypes, that major developmental delay could be observed, and that retinal changes were not universal. Mitral valve prolapse and mental impairment have only been reported previously [1].

Sacsin molecular chaperone protein (*SACS*) was discovered to be a key component of chaperone networks, folding, prevention of misfolding, and clearance of protein aggregates. *SACS* is highly expressed in the central nervous system, particularly in the brain and cerebellar Purkinje cells [11]. More than 200 mutations in *SACS* have been reported to cause neurodegenerative diseases, particularly ARSACS. Most mutations are located in exon 10, which interferes with interactions with Hsp70, disturbing the molecular chaper-one networks with a reduced function and causing neurodegenerative consequences [12]. Furthermore, decreased SACS levels would cause reduced chaperone activities, and the increased toxicity of GFP-ataxin-1[82Q] was confirmed to be associated with spino-cerebellar ataxia [13].

In this study, we identified a novel heterozygous *SACS* mutation (p.Val1335Ile) in a patient in their 50s through whole-exosome sequencing (WES). The patient presented atypical symptoms of ARSACS and a late onset, worsening rapidly. Interestingly, decreased mRNA expression and protein concentrations are associated with loss of function in sacsinopathy from haploinsufficiency. Furthermore, the patient in this study suggested the possibility of late-onset ARSACS with diverse symptoms and a wide range of disease onsets.

## 2. Materials and Methods

### 2.1. Case Report

A patient in their 50s presented to the Seoul National University Bundang Hospital in February 2020 with progressive weakness in his left hand (Table 1). The patient initially noticed this discomfort in the left arm 6 months prior to the initial hospital visit. Abnormal motor symptoms become apparent during activities, such as fastening shirt buttons or playing the piano. Over the course of 6 months, these symptoms gradually worsened, and the patient complained of difficulty with fine motor control, particularly when attempting to grasp objects.

They had no family history of neurodegenerative or genetic disorders. The patient exhibited normal intellectual abilities and held a general office job, with no exposure to metallic materials or similar substances. The patient had a history of strabotomy surgery 38 years ago, and a cyst was incidentally discovered during a brain MRI examination conducted 20 years ago while being evaluated for an episode of headache. The patient was taking medications for well-controlled type 2 diabetes.

**Figure 1:**
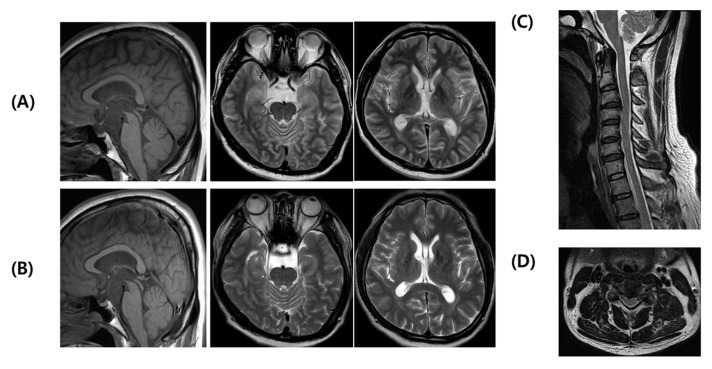
MRI images of the patient with Val1335IIe. (**A**) 3 months after onset and (**B**) 8 months after onset of brain MRI. Sagittal T1-weighted image showing an arachnoid cyst in the suprasellar-interpeduncular cistern displacing the third ventricular flow upward ((**A**)-Left). T2-weighted images in the pons ((**A**)-Middle) and around thalami ((**A**)-Right) exhibit no evidence of cerebellar atrophy. The serial MRIs show no change of the cerebellar peduncles ((**B**)-Left), pons ((**B**)-Middle), and thalami ((**B**)-Right). (**C**,**D**) The MRI of cervical spine. The sagittal and axial T2-weighted images presenting central canal stenosis (prominent at C3/4 level) without atrophy of the cervical spinal cord.

Various tests were performed on admission to our hospital. Examination revealed exotropia without any abnormal movement in the left eye. Systemic examination revealed no abnormalities, and pes cavus or hammer toes were not observed. Neurological examination revealed spastic rigidity and dysmetria in the left limb. The motor power of the left hand was assessed as Medical Research Council grade IV; otherwise, it was grade V, and sensory functions were normal. Increased deep tendon reflexes, clonus at the ankle, and extensor responses to plantar stimulation were detected in the left limb, indicating pyramidal tract signs. Laboratory tests conducted during the initial visit, including the assessment of vitamin levels and Lyme disease, showed no abnormalities. The patient had previously undergone brain and cervical spine MRI at another hospital in November 2019, 3 months before his first visit. Brain MRI revealed an arachnoid cyst in the suprasellar-interpeduncular cistern displacing the third ventricular flow upward (Figure 1A). C-spine MRI indicated grade 2 central canal stenosis at C3/4 (Figure 1C,D). A follow-up brain MRI in April 2020 showed no change in the cystic lesion, and no signs of brain atrophy were observed (Figure 1B). Electrodiagnostic testing, including normal nerve conduction studies and needle electromyography, showed no evidence of neuromuscular disorders. The fundus examination results were normal, and the patient was referred to an ophthalmologist to rule out Wilson’s disease and other eye problems. Eye examination and optical coherence tomography scans were normal.

After discharge, the patient continued to attend outpatient appointments and was closely monitored. The patient developed dysarthria and mild dysphagia 5 months after the initial visit to our hospital. One year after the first visit, spastic rigidity slowly progressed from the left side to both sides, accompanied by mild muscle atrophy. Neurological examination of the upper motor neuron signs revealed clonus in both ankles, extensor response in the Babinski sign, and thumb flexion in the Hoffman sign in both limbs. Sixteen months after the initial appearance of the symptoms, the patient became bedridden and was transferred to a nursing hospital. Tragically, the patient succumbed to complications resulting from COVID-19 infection in December 2021. Autopsy was not conducted in accordance with the wishes of the bereaved family. Genetic analysis of the family members was not pursued as they all declined, preventing the characterization of de novo or inherent mutations.

### 2.2. Genetic Analysis

DNA extraction was performed using a commercial kit (QIAamp DNA Blood Maxi Kit, #51194) according to the manufacturer’s protocol. The DNA sample was sent for WES (Novogen Inc., Beijing, China) according to the quality control criteria. Primers were synthesized (Bioneer Inc., Daejeon, Republic of Korea) using Primer3Plus (https://primer3plus.com/; accessed on November 2020). Specific primers were used for confirming *SACS* Val1335IIe: forward primer 5′-ATTTCCATGGGTTTGGACTG-3′; reverse primer 5′-AGCATTCGTGAATTGGCTTC-3. ’ The PCR samples were prepared with 2 μL DNA, 12 μL PCR master mix buffer (2X Hot Taq Master Mix, #ET16250. P), 5 μL (Betaine solution, #B0300), 4 μL distilled water, and 0.5 μL of each primer. The PCR cycles proceeded under the following conditions: 95 °C for 3 min for one cycle, 95 °C for 40 s, 55.5 °C for 40 s, 72 °C for 40 s for 32 cycles, and 72 °C for 3 min for one cycle. After PCR, its products were isolated in 1.5% agarose gel, and images were obtained using the Davinch-Chemi™ Imaging system (Davinch-K, Seoul, Republic of Korea). Sanger sequencing was performed to validate the variants (Bioneer Inc., Daejeon, Republic of Korea).

### 2.3. In Silico Analysis

Bioinformatics analyses of missense mutations were performed using Polyphen2 (http://genetics.bwh.harvard.edu/pph2; accessed on November 2020), SIFT (http://sift.jcvi.org/; accessed on November 2020), Mutationtaster (http://www.mutationtaster.org; accessed on November 2020), and PROVEAN (http://provean.jcvi.org/index.php; accessed on November 2020) for the pathogenic relations. For protein 3D modeling, the structure was predicted using the RaptorX web server (http://raptorx.uchicago.edu/; accessed on November 2020) and analyzed using BIOVIA Discovery Studio Visualizer (BIOVIA, San Diego, CA, USA).

### 2.4. Assessment of Protein Concentrations and mRNA Expression

Plasma was isolated after centrifuging whole blood samples at 800× *g* for 30 min and was stored at −80 °C until use. The concentration of sacsin was measured using a commercial kit (Human Sacsin (SACS) ELISA Kit, #MBS9312451) with a heparinized plasma sample. Briefly, the sample (50 μL) and HRP-conjugate reagent (100 μL) were added to the antibody-SACS-coated plate and reacted at 37 °C for 1 h. After washing the plate four times with a wash buffer, the chromogen solutions A (50 μL) and B (50 μL) were added and reacted at 37 °C for 15 min. Stop solution (50 μL) was added, and the optical density at the 450 nm wavelength was measured using a VICTOR 3™ multi-spectrophotometer (PerkinElmer, Waltham, MA, USA).

mRNA was extracted from the whole blood (AccuPrep^®^ Blood mRNA Extraction Kit, #K-3144) following the manufacturer’s manual. The extracted mRNA was synthesized into cDNA (AccuPower™ RocketScript™ Cycle RT PreMix & Master Mix, #K-2201) and stored at –20 °C until use. Primers for RT-PCR were synthesized by Bioneer Inc. (Daejeon, Republic of Korea). *SACS* primers were used for confirming the *SACS* mRNA level: forward primer 5′-GGTCTGTGGTTCAATAGAGGAG-3′; reverse primer 5′-GTTTGCTTTCTTGTTCACTGAG-3′. Beta-actin primers were used for normalizing *SACS* mRNA levels: forward primer 5′-AGAAAATCTGGCACCACACC-3′; reverse primer 5′-GGGGTGTTGAAGGTCTCAAA-3′. RT-PCR was performed using SYBR green (AccuPower^®^ 2X GreenStar™ qPCR Master Mix, #K-6251), following the manufacturer’s manual.

### 2.5. Statistical Analysis

Graphs were drawn using GraphPad Prism software version 8.0.8 (GraphPad Software Inc., San Diego, CA, USA). Compared with healthy age-matched controls (*n* = 3, protein measurement; *n* = 5, mRNA expression), it was difficult to perform a statistical analysis between the groups because there was only one patient (*n* = 1). Accordingly, the mean and standard deviation of the measured values of the patients and healthy age-matched controls were calculated.

## 3. Results

### 3.1. Genetic Findings

**Figure 2:**
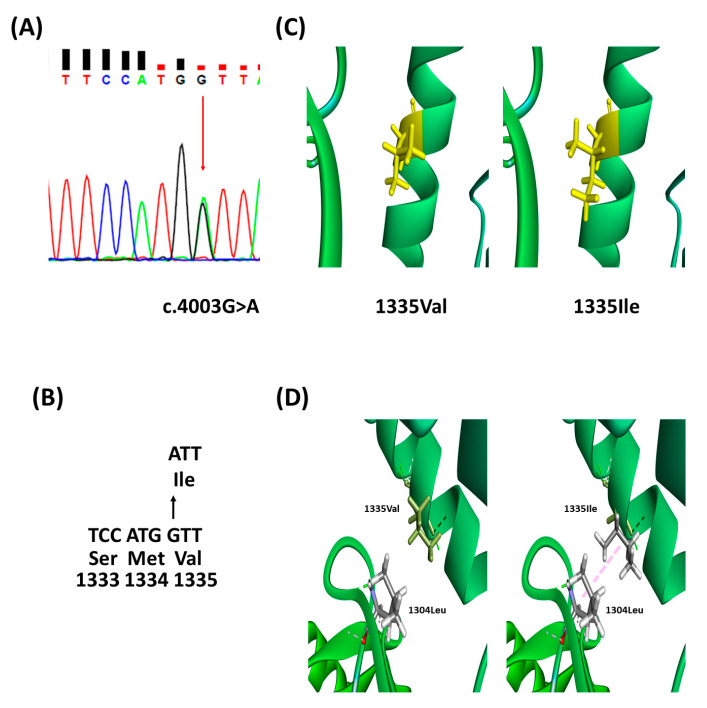
In vitro results of *SACS* Val1335Ile. (**A**) Sanger sequencing confirmed change of c.4003G>A in patient and (**B**) change of amino acid Val to Ile on *SACS* 1335 location. Arrows indicate a change of nucleotide G to A, leading to the change of Val to Ile. (**C**) 3D modeling predicted *SACS* 1335Val (Wild-type) and 1335Ile (Mutant). The yellow part shows the amino acid change from Val to Ile. (**D**) It could interact with 1304Leu, as amino acids changed from Val to Ile in *SACS* 1335 position.

A novel heterozygous mutation in *SACS* (c.4003G>A, p.Val1335Ile) in exon 10 was identified via WES and verified via Sanger sequencing (Figure 2A,B), which was not found in 1000 Genomes and EXAC databases. The pathogenicity of *SACS* Val1335Ile indicated a damaging variant in the prediction software (Table 2).

**Figure 3:**
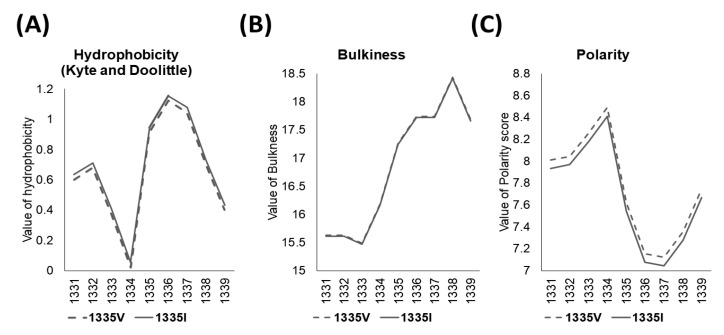
ExPASY scores of *SACS* Val1335Ile compared to wild-type. The results indicated (**A**) hydrophobicity scores (Kyte and Doolittle), (**B**) bulkiness, and (**C**) polarity scores on change of Val to Ile.

The ExPASY tool confirmed the hydrophobicity (Kyte and Doolittle), bulkiness, and polarity scores of amino acid residues near the mutation. Mutation from Val to Ile caused an increase in the hydrophobicity score from 0.911 to 0.944. The bulkiness and polarity scores (Grantham) decreased from 17.26 to 17.241 and 7.622 to 7.544, respectively (Figure 3). The prediction of the protein structure showed that the change of *SACS* 1335 Val to Ile added new alkyl interactions with the 1304Leu residue, which may cause the rotation of the helical structure (Figure 2C,D).

### 3.2. Measurement of Protein and Gene Expression

**Figure 4:**
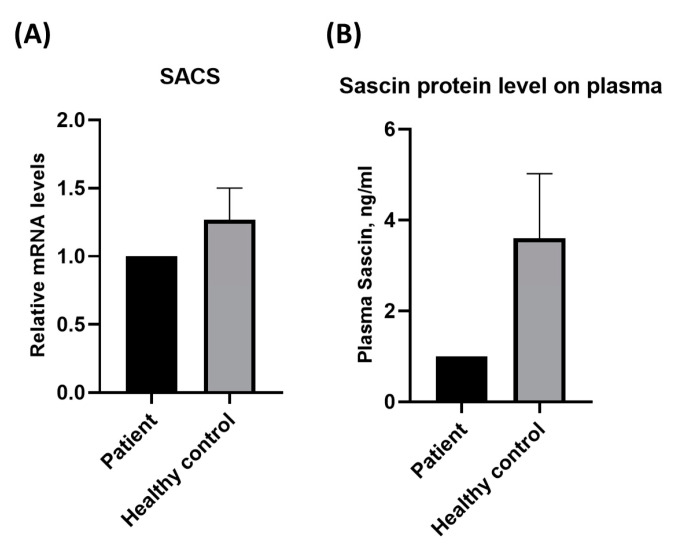
Difference of *SACS* gene expression and protein level according to Val1335Ile. (**A**) *SACS* gene expressions and (**B**) Sacsin protein levels were decreased in the patient compared to those in healthy age-matched controls, supporting haploinsufficiency.

The *SACS* gene had a relatively low mRNA expression, according to the real-time PCR (RT-PCR) results (Figure 4A). ELISA to measure sacsin protein levels showed a decrease in the patient relative to healthy age-matched controls (Figure 4B).

## 4. Discussion

In the present study, a patient in their 50s presented with progressive clumsiness and abnormal motor movement symptoms. As the symptoms worsened rapidly, a novel *SACS* mutation (c.4003 G>A, p.1335 Val>Ile, Heterozygous) was discovered using WES, although no pathogenic mutations with low frequency were found in ataxia-related genes, such as SPG7, FXN, and GFAP. Based on in vitro analysis, *SACS* Val1335IIe was significant as a possible disease-causing gene (Table S1). *SACS* Val1335Ile was predicted to be damaged by the pathogenicity predictive algorithms in Polyphen-2 and MutationTaster (Table 2), where additional alkyl interactions are generated from Val to Ile through the 3D modeling prediction of increased hydrophobicity for tight hydrophobic packaging.

Clinically, the patient exhibited progressive spastic rigidity, spasticity, and cerebellar ataxia without peripheral neuropathy. Although electrophysiological tests appeared normal in the early stages, brain MRI consistently revealed linear hyperintensities in the pons and cerebellar atrophy, particularly in the upper vermis, on T2 and FLAIR images. The patient complained of dysarthria and dysphagia without any additional characteristics of ARSACS. The onset of symptoms in patients in their 50s represents an unusual case of ARSACS that typically manifests at a very young age. However, our findings are similar to those of late-onset cases reported in other studies, including those from Belgium, emphasizing the variability in ARSACS symptoms and onset. Atypical features, such as altered reflexes, Babinski’s sign, muscle atrophy, and optic atrophy, further complicate the clinical presentation of late-onset patients, suggesting a broad spectrum of sacsinopathy through *SACS*, especially in heterozygous cases [10,14,15,16,17,18]. The patient showed late clinical symptoms with faster deterioration than other late-onset patients, which could be due to the heterozygous mutation and delayed disease onset compared to patients with homozygosity [3]. Interestingly, our study confirmed that sacsin concentrations decreased compared to that in age-matched controls, and the patient’s *SACS* gene expression was also reduced, correlating with decreased sacsin levels, supporting haploinsufficiency. In general, *SACS* variants are known to induce loss of function, including protein expression and calcium homeostasis regulation [19,20,21,22,23,24], and the sacsin protein level is reduced in heterogeneous *SACS* mouse models [19]. Hence, these decreases in both mRNA and protein levels could support the genetic importance of loss-of-function, which could influence disease pathology. In addition, our results revealed that *SACS* Val1335Ile is a pathogenic gene in patients with sacsinopathy, although the patient had clinical features that were slightly different from those of typical ARSACS.

The study had limitations insofar as an autopsy could not be performed, and bio-sample experiments could not be conducted due to the patient’s death from COVID-19. Nevertheless, expression measurements in patients with late-onset ARSACS were the first to measure a mutation-induced reduction in order to support gene discovery. It may be possible to further prove the pathogenicity of the mutation identified in this study by generating a mutated cell line.

## 5. Conclusions

In conclusion, we identified a case of adult-onset sacsinopathy with a novel heterozygous *SACS* mutation (c.4003 G>A, p.1335 Val>Ile). Although the disease onset was late and did not correspond to all classic clinical ARSACS characteristics, pathogenicity prediction indicated probable damage. Furthermore, sacsin protein and mRNA levels were reduced, indicating genetic pathogenicity from haploinsufficiency. A comparison with other late-onset cases suggests that ARSACS in terms of the heterozygosity status may be a disease with a wider spectrum and variety of symptoms [8,25]. The production of mutant cells in future functional studies will help determine the disease mechanism of this gene in detail.

## Figures and Tables

**Table 1:** Clinical characteristics of the case.

Patient	Age/Sex	Age of Onset	Clinical Symptoms at Presentation	Cerebellar Ataxia	Spasticity	Polyneuropathy	Muscle Weakness/Atrophy	Ambulation	Brain MRI	Cervical Spine MRI
1	51/M	51	Weakness in the left distal upper limb	Yes	Yes	No	Progressed from left to both sides	Wheelchair-bound since age 53	Arachnoid cyst, otherwise normal	Central canal stenosis in C3/4

**Table 2:** Prediction of pathogenicity of *SACS* Va1335Ile. The pathogenicity of *SACS* Val1335Ile was predicted by algorithms, resulting as probable pathogenicity.

Predictive Algorithms	Score	Judgment
MutationTaster	1.000	Disease-causing
Polyphen-2 (Hum VAR)	0.858	Probably damaging
PROVEAN	−0.322	Neutral
SIFT	0.12	Tolerated

## Data Availability

Data are contained within the article and supplementary materials.

## Supplementary Materials

The following supporting information can be downloaded at: https://www.mdpi.com/article/10.3390/cimb45120619/s1. Supplementary Table S1. Ataxia-related genes in patient. Ataxia-related genes were predicted its pathogenicity and its results did not be pathogenic. This means that these genes are difficult to see as disease-causing genes.
