# Supplementary material for: A Novel Mutation in Sacsin, p.Val1335IIe, May Cause Late-Onset Sacsinopathy Due to Haploinsufficiency
Source: Curr Issues Mol Biol. 2023 Dec 9;45(12):9917–25. doi: 10.3390/cimb45120619 (PMC10741900; doi:10.3390/cimb45120619)
Supplement: Supplementary file 1 [file cimb-45-00619-s001.zip › cimb-2765337-supplementary.pdf]

Supplementary Table S1. Ataxia-related genes in patient. Ataxia-related genes were predicted its pathogenicity and its results did not be pathogenic. This means that these genes are difficult to see as disease-causing genes.

Gene	Disorder	Mutation	SIFT / Polyphen-2	Frequency (1000g_EAS)
ATXN3	Machado-Joseph Disease	rs1048755 (c.634G>A, p.V212M)	Tolerated / Benign	0.4405
		c.67_68insCAGCAGCAGCAGCAGCAGCAGCAGCAGCAG	NA	NA
FXN	Friedreich ataxia	synonymous SNV	-	-
GFAP	Alexander Disease	rs9916491 (c.1276A>G, p.T426A)	Tolerated / Benign	0.251
		rs1126642 (c.883G>A:p.D295N)	Damaging / Damaging	0.1359
KIF1A	Spastic paraplegia 30	synonymous SNV	-	-
		rs10594016 (c.2751_2753del/p.917_918del)	NA	NA
KIF1C	Spastic Ataxia 2	synonymous SNV	-	-
MARS2	Spastic Ataxia 3	-	-	-
MTTP	Abetalipoproteinemia	rs2306986 (c.294G>C, p.E98D)	Tolerated / Benign	0.5143
		rs3792683 (c.497AG, p.N166S)	Tolerated / Benign	0.5143
		rs2306985 (c.891C>G, p.H297Q)	Tolerated / Benign	0.6706
SPART	Troyer syndrome	-	-	-
SPG7	Spastic paraplegia 7	-	-	-
TTPA	Autosomal recessive ataxia with vitamin E deficiency	-	-	-
